# Full robotic whole graft liver transplantation: Is a new era coming?^☆^

**DOI:** 10.1016/j.iliver.2024.100128

**Published:** 2024-11-06

**Authors:** Yipeng Pan, Jimin Liu, Qi Ling

**Affiliations:** a Department of Surgery, The First Affiliated Hospital, Zhejiang University School of Medicine, Hangzhou 310003, China; b Department of Transplantation, The Second Affiliated Hospital of Hainan Medical University, Haikou 570311, China; c Department of Pathology and Laboratory Medicine, Mt Sinai Hospital, 600 University Ave, Toronto M5G 1X5, ON, Canada

With great interest, we read the article conducted by Pinto-Marques H et al., entitled “Full Robotic Whole Graft Liver Transplantation: A Step Into The Future” [1], which was published in the current issue of *Annals of Surgery*. In this article, six patients receiving full robotic whole liver transplantation (RLT) were reported. All 6 cases of RLT were performed successfully without severe post-operative complications during a median follow-up time of about three months. The advantages offered by RLT seem to have less impact on the abdominal wall, with reduction of postoperative pain and fast recovery. Therefore, the authors conducted an incredible minimally invasive surgery in liver transplantation (LT). We should congrats the authors who have made a milestone in the surgical technique of LT.

In the past decades, minimally invasive concept has been widely accepted by surgeons. The laparoscopic approach was soon adapted in the acquisition of donor graft in living donor LT. To date, pure laparoscopic donor left lateral sectionectomy has been validated for its safety and advantages and has become the standard in experienced centres [2]. Compared to the laparoscopic approach, the robotic one could broaden the indications for minimally invasive surgery due to its technical advantages. The robotic surgical system overcomes the reduced visualization, restricted range of motion and physiological tremor associated with laparoscopic surgery and allows for a comparatively easier transition from technical feasibility to reproducibility [3].

As compared to the donor graft acquisition, graft implantation is a much more complex procedure, which makes a great challenge for minimal invasive approach. It is not surprised that RLT largely increases the operation time (∼3-folds). More importantly, RLT increases graft warm ischemia time (GWIT), which ranges between 55 and 90 min [1]. In contrast, GWIT is about 19–51 min in the classical open approach [4]. GWIT is a well-known risk factor for various post-transplant complications such as biliary stricture, because biliary epithelial cells are more susceptible to injury by warm ischemia and hypoxia/reoxygenation compared with the hepatocytes [5]. Therefore, in the current stage, RLT has major shortcomings, particularly the increased ischemia injury, which may cause adverse prognosis in the long-term follow up. Although the authors did not mention this in the article, we believe it would be very hard to deal with intra-operative complications such as hemorrhage as well. It could be challenging to quickly and exactly identify the cause, site, and severity of bleeding due to the reduced view of laparoscopy and less accessible for emergency responses. In addition, RLT still requires an abdominal incision to remove the diseased organ and implant the liver graft through an upper midline or periumbilical incision of sufficient length (8–15 cm according to the explant and graft size). Furthermore, RLT increases the cost. We roughly compared the RLT with classical LT in Table 1.

**Table 1:** Advantages comparison of RLT and classic LT.

Operation	Operative time	GWIT	Cost	Postoperative pain	In-hospital stay
RLT	Longer	Longer	Higher	Less	Shorter
Classical LT	Shorter	Shorter	Lower	More	Longer

RLT: full robotic whole liver transplantation.

LT: liver transplantation.

GWIT: graft warm ischemia time.

Comparing to the classical LT, RLT has longer operative time, GWIT and higher cost, as well as shorter hospital stay and less postoperative pain.

However, from our point of view, there are issues with much greater significance than pursuing minimal invasive surgical technique in the field of LT. These issues could be surgical technique and non-surgical technique-related.

## 1 In surgical technique

Split liver transplantation (SLT) can effectively expand the donor pool. Vascular segmentation and reconstruction are the key techniques. It is mandatory but difficult to ensure sufficient blood inflow and outflow (especially Segment V and VIII venous reconstruction) and reduce complications such as small-for-size syndrome.

Portal vein thrombosis (PVT) is not a contraindication of LT. However, extensive or complex type of PVT such as cavernous transformation of the portal vein (CTPV) significantly increases the surgical difficulty. Meso-Rex shunting, insertion of portal vein stent (PVS) and modified transjugular intrahepatic transjugular intrahepatic (mTIPS) might provide solutions, which could reduce the need for LT or transform contraindications of LT into indications.

Now, Cavo-portal hemitransposition, reno–portal anastomosis (RPA) in the absence of spleno-renal shunt (SRS), and PV arterialization were categorically excluded from any surgical plan. Patients with a preoperative diagnosis of complete obstruction of the entire superior mesenteric vein (SMV) without a portal-systemic shunt (PSS) were referred for multivisceral transplantation (MVT). We believe that the implementation of innovative techniques, which over time will prove to be increasingly safe and effective [6].

Recurrence of liver cancer after LT remains a major challenge. Both our team and others have made great efforts including surgical techniques and perioperative management to reduce the risk of cancer recurrence. We believe the “no-touch principle”, which means en-bloc total hepatectomy including the retrohepatic inferior vena cava (IVC) and all 3 hepatic veins (HVs), could minimize the possibility of liver cancer cell spread [7].

The sequence for revascularization of the portal vein and hepatic artery has been debated over decades referring to its impacts on the prognosis particularly biliary stricture. Research concluded that the contemporaneous portal and hepatic artery (CPA) protocol can decrease the incidence of biliary complications substantially after LT. This effect was evident on nonanastomotic strictures but not on anastomotic strictures [4].

Acute-on-chronic liver failure (ACLF) is one of the major indications of LT. However, patients with ACLF are associated with a poorer prognosis as compared with non-ACLF patients probably due to multi-organ failure. The combination of organ replacement technology, such as artificial liver support and extracorporeal membrane oxygenation (ECMO) with LT, has been reported to improve the post-transplant prognosis in these sickpatients.

## 2 Beyond surgical technique

There is a consensus that the long-term stability of graft function is crucial to the prognosis and quality of life of the recipient [8]. However, most liver recipients need lifelong immunosuppression drugs, although liver is an immune privilege organ. The maintenance of calcineurin inhibitors and/or mTOR inhibitors causes various adverse effects, like infection, chronic kidney diseases, tumor, metabolic disorders and cardiovascular attack. Therefore, effective and low toxic drugs for immunosuppressive treatment are needed. Nanoparticle-based delivery of immunomodulators provides potential precision strategy, which can reduce drug systemic toxicity [9]. Moreover, recent studies using current advanced technology has made a big step for uncovering the mystery of immune tolerance in orthotopic mouse LT model, which is a unique tolerance model as compared to other organ transplantation (heart, kidney, skin, etc) [10]. Inducing immune tolerance using cell therapy such as Tregs, bone marrow-derived mesenchymal stem cells (MSCs) and regulatory dendritic cells (DCs) has launched phase I or Phase II clinical trials.

To address the organ shortage, liver grafts with advanced age, significant steatosis, or from donors with circulatory death are being used. The marginal grafts, however, could result in an increased risk of graft-associated complications, such as primary nonfunction, delayed graft function and late biliary injuries. As potential solutions, machine perfusion technologies and ischemia-free LT have been applied to improve outcomes. Such innovation could offer a reliable platform for graft preconditioning and repairment. However, the high cost and the technical complexity limited the widely use of machine perfusion.

In summary, RLT can be considered as a milestone in minimal invasive surgery history. However, we should pay more attention in the issues with better clinical value and provide beneficial in both long-term survival and life quality.

## Funding

This research received no external funding.

## CRediT authorship contribution statement

**Yipeng Pan:** Writing – original draft. **Jimin Liu:** Writing – review & editing. **Qi Ling:** Writing – review & editing.

## Acknowledgments

None.

## Declaration of competing interest

The authors declare no conflict of interest.

## Data availability statement

Data will be made available on request.

## Ethics statement

Not applicable.

## Informed consent

Not applicable.
